# The ferumoxytol in renal insufficiency study (FiRST)

**DOI:** 10.1186/1532-429X-15-S1-P228

**Published:** 2013-01-30

**Authors:** Sreekanth Vemulapalli, Lucien Abboud, Cassidy Duran, Igor Klem, Han W Kim, Anna Lisa Crowley, Miguel A Quinones, Faisal Nabi, John P Middleton, William A Zoghbi, Raymond J Kim, Dipan J Shah

**Affiliations:** 1 Duke Cardiovascular Magnetic Resonance Center, Duke University, Durham, NC, USA; 2 Methodist DeBakey Heart and Vascular Center, Methodist DeBakey Hospital, Houston, TX, USA; 3 Nephrology, Duke University, Durham, NC, USA; 4 Cardiology, St. Francis Heart and Vascular Center, Topeka, KS, USA

## Background

Imaging in patients with renal insufficiency (RI) is limited by contrast induced nephropathy and nephrogenic systemic fibrosis. Ferumoxytol is an FDA approved superparamagnetic iron compound used for the treatment of anemia in patients with renal insufficiency. Previous studies of its use as an MRA contrast agent did not assess safety, were not conducted in patients with RI, and were limited by small sample size. We characterized the safety, image quality, and clinical impact of ferumoxytol-enhanced MRA (FeMRA) in patients with RI as compared to gadolinium-enhanced MRA (GeMRA) in patients without RI.

## Methods

All patients referred for cardiovascular MRA at two institutions between June 2009 and February 2012 were entered into a prospective database. Patients with chronic RI, defined as GFR < 30 ml/min, or acute kidney injury were eligible for FeMRA. Prior to FeMRA, patients underwent screening for iron overload via history and serum iron studies or T2* imaging. Images were assessed quantitatively and qualitatively on a 1-5 Likert scale by two blinded readers. Three-month follow-up for adverse events, further imaging, and subsequent endovascular or surgical interventions was obtained. A comparator group was chosen at random from patients without RI who underwent GeMRA for similar indications during the same time period (n=70).

## Results

Of 6198 patients undergoing MRA, 203 were eligible for FeMRA. Of these, 7 patients were excluded due to elevated ferritin or low T2*, 2 did not provide consent for FeMRA, and 39 underwent GeMRA based on attending preference. Of 155 FeMRA scans, symptomatic adverse events (IV site reaction, nausea/vomiting) occurred in 1.3% of FeMRA, and 0.4% had symptomatic hypotension requiring IV fluid administration. Subjective image quality was similar between FeMRA and GeMRA (4.6 ± 0.6 [n=155] vs. 4.6 ± 0.6 [n=70], p = 0.72). Contrast-to-noise ratio was also indistinguishable between FeMRA and GeMRA (45.2 ± 41.6 [n=30] vs. 45.9 ± 23.8 [n=18], p=0.94). FeMRA and GeMRA were of sufficient diagnostic quality to prevent further imaging in 76.2% and 70% of patients, respectively (p=0.12; 95% CI of odds ratio 0.86 - 3.62). FeMRA and GeMRA guided subsequent endovascular or surgical intervention in 33.5% and 42.9% of patients, respectively (p=0.23; 95% CI of odds ratio 0.78 - 2.75).

## Conclusions

FeMRA has an acceptable safety profile, excellent image quality, and impacts clinical management in patients with RI similarly to GeMRA in patients with preserved renal function. Ferumoxytol represents a currently available and potentially effective MRA contrast agent for patients with RI.

## Funding

This work was funded internally by the Duke Cardiovascular Magnetic Resonance Center. No relevant industry or government funding relationships exist.

**Figure 1:**
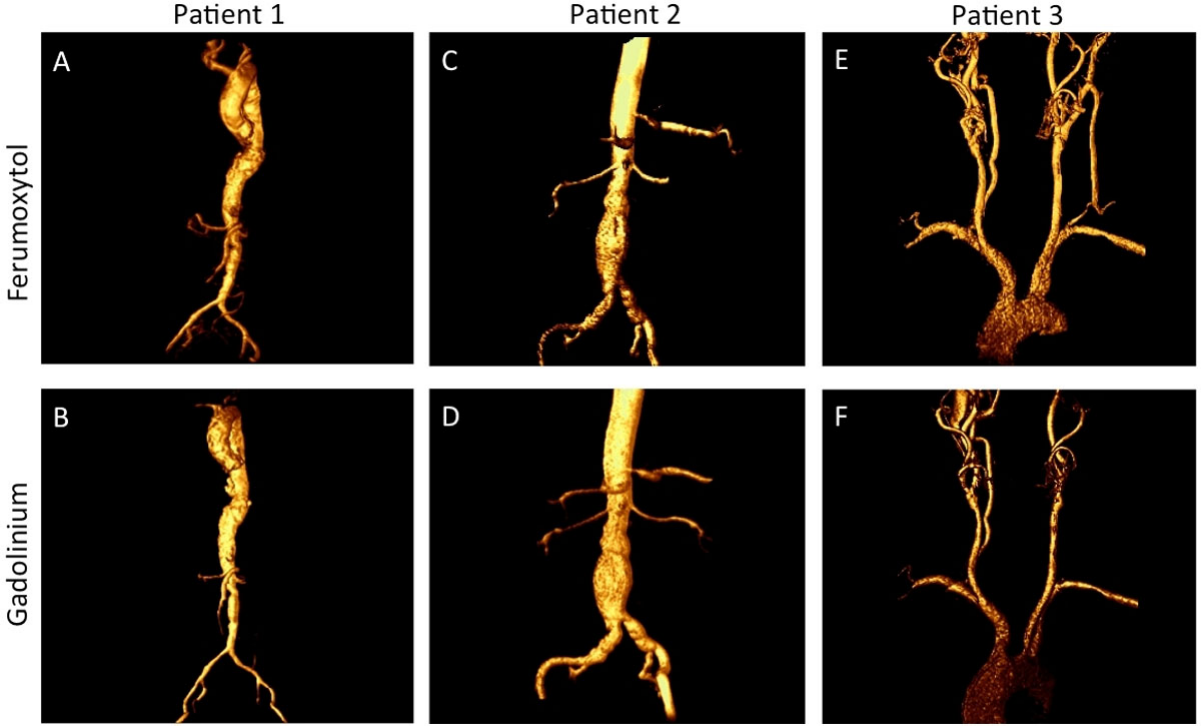
Ferumoxytol thoracoabdominal MRA (A) in a patient with GFR < 30 and gadolinium thoracoabcominal MRA (B) in the same patient 2 months earlier. Ferumoxytol abdominal aortic MRA in a patient with GFR < 30 (Panel C) and gadolinium abdominal aortic MRA in the same patient 2 months earlier (Panel D). Ferumoxytol carotid MRA (E) and gadolinium carotid MRA (F) in the same patient 1 year earlier.

